# Immune checkpoint inhibitor-induced inflammatory arthritis as a potential biomarker for long-term response to tislelizumab in advanced head and neck carcinoma: a case report and literature review

**DOI:** 10.3389/fonc.2026.1772943

**Published:** 2026-04-13

**Authors:** Xiaoqiong Shi, Jianqiao He, Guoning Yu, Yingna Gao, Yi Ma, Hongliang Zheng, Minhui Zhu, Caiyun Zhang

**Affiliations:** Department of Otolaryngology-Head and Neck Surgery, Changhai Hospital, Naval Medical University, Shanghai, China

**Keywords:** head and neck squamous cell carcinoma, immune checkpoint inhibitor, immune checkpoint inhibitor-induced inflammatory arthritis, immune-related adverse events, tislelizum

## Abstract

**Background:**

Immune checkpoint inhibitors (ICIs) have emerged as promising treatment strategies in cancer immunotherapy. However, a significant proportion of patients do not respond to these therapies, underscoring the need for reliable biomarkers of treatment responsiveness. Although a potential correlation between the development of immune-related adverse events (irAEs) and favorable response to ICI therapy has been demonstrated, the predictive reliability of irAEs for treatment efficacy remains to be fully established.

**Case summary:**

This report presents a case of advanced unknown primary carcinoma of the head and neck (UPCHN) with rapid disease progression following initial treatment with neck dissection and concurrent chemoradiotherapy. Subsequent administration of a weekly regimen comprising paclitaxel, carboplatin, and cetuximab (PCC) resulted in complete response (CR). Maintenance therapy with cetuximab and tislelizumab was then initiated. After 20 months of sustained CR, the patient developed grade 3 ICI-induced inflammatory arthritis (ICI-IA), necessitating discontinuation of ICI therapy. Notably, the patient remained in complete remission for over 1 year following cessation of ICI therapy, with no clinical or radiographic evidence of tumor progression.

**Conclusion:**

This case suggests that the development of ICI-IA reflects an immune hyperactivated state that correlates with long-term antitumor response to tislelizumab. The sustained remission observed in this patient following ICI discontinuation highlights the potential role of irAEs, particularly ICI-IA, as a potential biomarker of ICI efficacy. Further prospective, large-scale studies are warranted to validate these findings and assess their clinical applicability.

## 1 Introduction

Immune checkpoint inhibitors (ICIs), particularly anti-programmed death receptor-1 (anti-PD-1) and anti-programmed death ligand 1 (anti-PD-L1) antibodies, function by blocking the PD-1/PD-L1 signaling axis, thereby enhancing cytotoxic T cell-mediated antitumor immunity. These agents have emerged as a promising strategy for tumor management due to their substantial clinical efficacy (1). However, a significant proportion of patients fail to respond to these therapies, highlighting the need for reliable biomarkers to predict treatment response. Studies on predictive biomarkers have predominantly focused on tumor signatures such as microsatellite instability-high status, tumor mutational burden, and PD-L1 expression levels (2, 3). However, the clinical applicability of these biomarkers remains limited due to the inherent spatial and temporal heterogeneity of tumors and the invasive nature of biopsy procedures required for their assessment (4). These limitations underscore the crucial need for more clinically accessible, non-invasive biomarkers capable of accurately reflecting systemic immunologic activity and predicting immunotherapeutic efficacy.

Immune-related adverse events (IrAEs) are reactions that occur when ICI-induced immune activation causes the immune system to aberrantly attack the body’s normal tissues and organs. These adverse events can affect multiple organ systems, with varying onset timing and severity. Although the precise immunopathogenesis of irAEs remains elusive, they are largely attributed to the off-target effects of activated T cells, consistent with the underlying mechanism of ICIs (5, 6). Several comprehensive meta-analyses (7–9) have consistently shown that patients who develop irAEs exhibit improved clinical outcomes, including enhanced progression-free survival (PFS), overall survival (OS), and objective response rate (ORR). These findings suggest that irAEs may serve as a potential biomarker of systemic immune activation and therapeutic responsiveness to ICIs. Although an association between irAEs occurrence and ICI treatment response has been established, critical clinical questions remain, particularly regarding the prognostic implications of irAEs characteristics such as site, severity, and timing of onset. A comprehensive understanding of these factors is essential to elucidate the immunologic mechanisms underlying ICI therapeutic efficacy and to refine patient selection and management strategies.

This report presents a case of advanced unknown primary carcinoma of the head and neck (UPCHN). The patient was treated initially with weekly paclitaxel, carboplatin, and cetuximab (PCC) chemotherapy, followed by maintenance therapy with cetuximab and tislelizumab after failure of neck dissection and chemoradiation. The patient achieved a CR and maintained excellent clinical performance (Karnofsky performance status [KPS] = 100). However, during the 20th month of maintenance therapy, the patient developed grade 3 ICI-induced arthritis (ICI-IA), necessitating the discontinuation of the ICI therapy. Notably, the patient remained in CR for over 1 year after cessation of the therapy, with no signs of tumor progression and a sustained PFS of over 36 months. A systematic literature review was also conducted to further explore the association between irAEs and the therapeutic efficacy of ICI.

## 2 Case presentation

**Figure 1:**
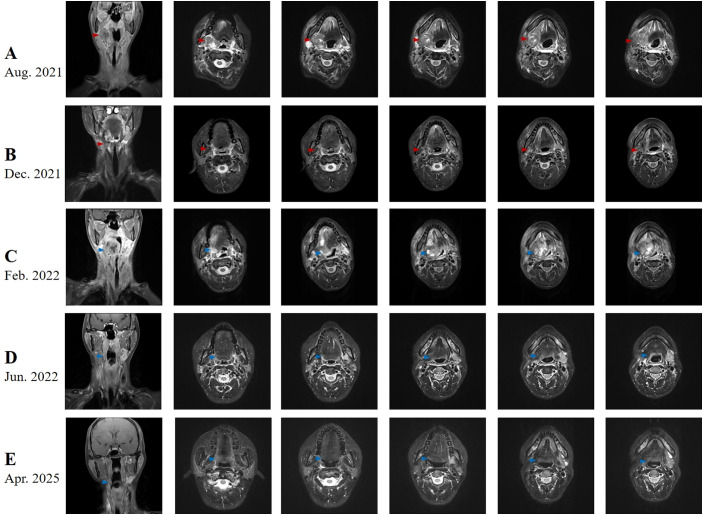
Radiographic imaging of advanced unknown primary carcinoma of the head and neck (UPCHN) during therapy and follow-up. **(A)** August 2021: Initial scan showing a right submandibular mass (red arrows). **(B)** December 2021: The disappearance of all target lesions (red arrows) following neck dissection and chemoradiotherapy. **(C)** February 2022: A newly developed irregular mass in the right oropharyngeal region (blue arrows). **(D)** June 2022: The disappearance of all target lesions (red arrows) after treatment with the weekly PCC regimen (blue arrows). **(E)** April 2025: Most recent MRI showing no signs of tumor recurrence or progression (blue arrows).

**Figure 2:**
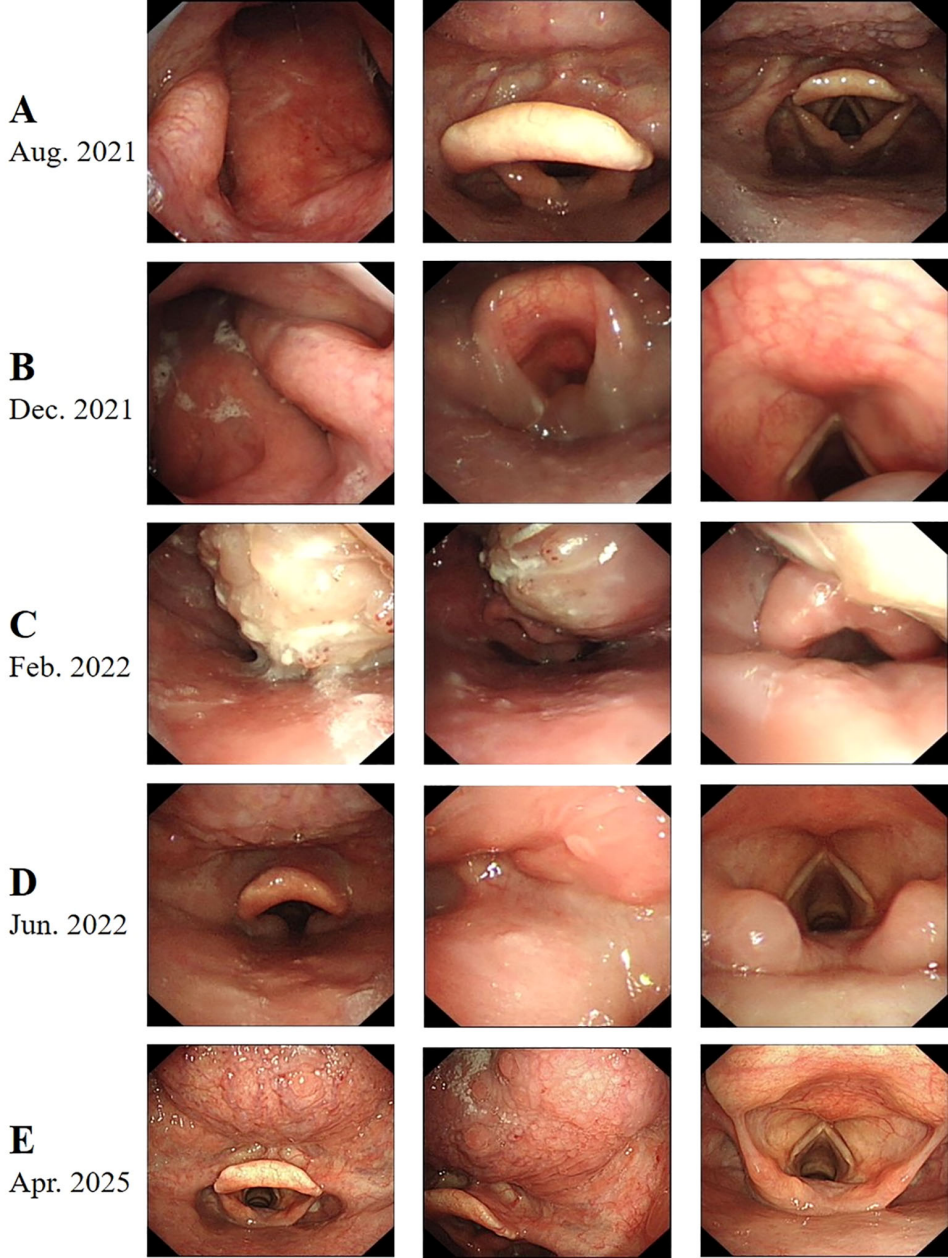
Fiberoptic laryngoscopy (FOL) images of recurrent/metastatic head and neck squamous cell carcinoma (HNSCC). **(A)** August 2021: Initial FOL showing no obvious abnormalities in the nasopharynx, tongue base, oropharynx, or hypopharynx. **(B)** December 2021: Complete response (CR) following neck dissection and chemoradiotherapy, based on RECIST criteria. **(C)** February 2022: Repeat FOL showing a mass on the right root of the tongue. **(D)** June 2022: CR after treatment with the weekly PCC regimen. **(E)** April 2025: Most recent FOL showing no signs of tumor progression.

**Figure 3:**
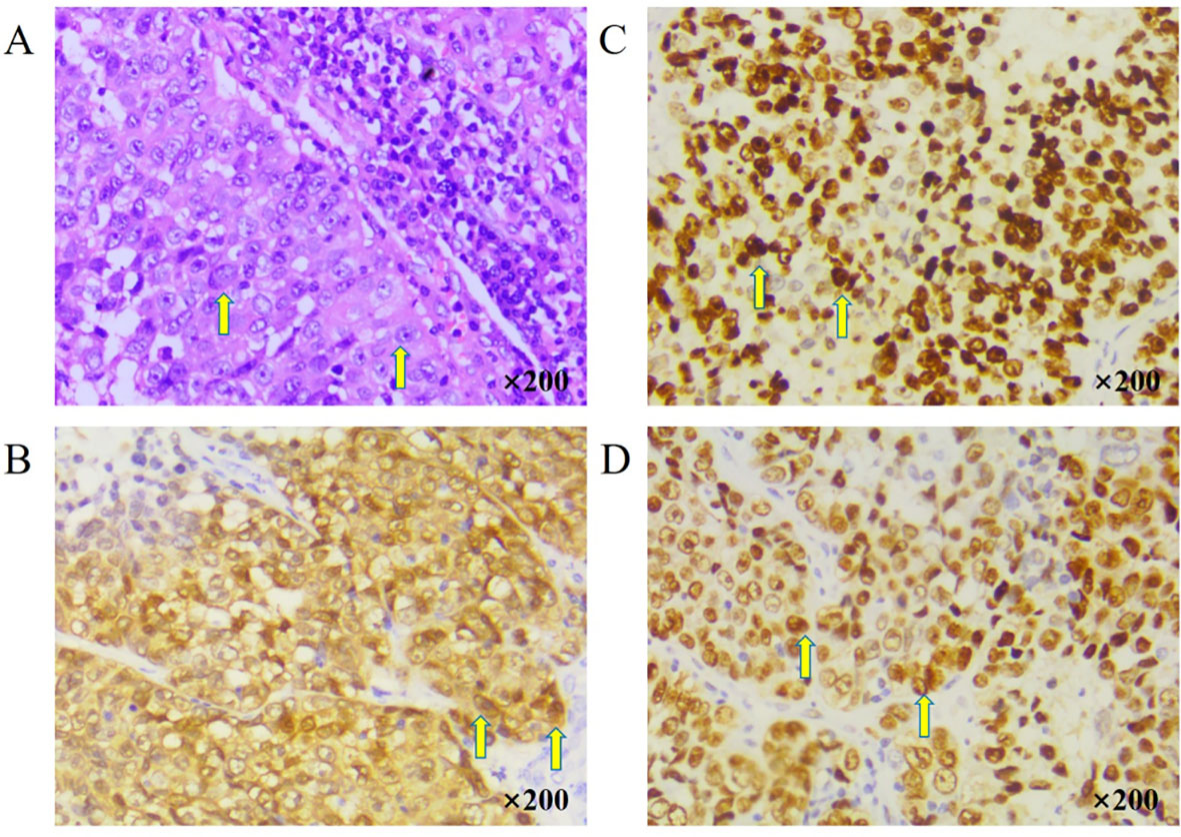
Histopathological and immunohistochemical examination of the right upper neck mass. **(A)** Hematoxylin and eosin (H&E) staining of the resected right upper neck mass (200x magnification). **(B)** Immunohistochemistry (IHC) showing positive p16 expression (200x magnification). **(C)** IHC showing high proliferative activity, indicated by Ki-67 expression in approximately 80% of tumor cells (200x magnification). **(D)** IHC showing p53 expression with a mutant-type expression pattern in approximately 90% of tumor cells (200x magnification).

A middle-aged patient presented to the hospital in August 2021 with a right upper neck mass measuring 50 mm × 60 mm. The patient reported no history of smoking, alcohol consumption, or cancer. Initial evaluation, including ultrasound (US), magnetic resonance imaging (MRI) (**Figure 1A**), fiberoptic laryngoscopy (FOL) (**Figure 2A**), and histopathologic examination, confirmed the diagnosis of metastatic squamous cell carcinoma of the head and neck. Notably, no primary tumor was identified on whole-body positron emission tomography–computed tomography (PET/CT), consistent with carcinoma of unknown primary. Following a comprehensive clinical assessment, the patient underwent neck dissection involving levels I, II, and III of the cervical lymph nodes. Histological examination of the resected tumor using hematoxylin and eosin (H&E) staining confirmed metastatic squamous cell carcinoma (**Figure 3A**). Immunohistochemistry (IHC) revealed that the tumor cells were positive for p16, Ki-67 (80%), p53 (90% mutant type) (**Figures 3B–D**), and p63 and negative for cytokeratin 7 (CK7), progesterone receptor (PR), estrogen receptor (ER), and transcription intermediary factor 1 (TIF1). Epstein–Barr encoding region (EBER) *in situ* hybridization was also negative. Due to the presence of extranodal extension, the patient received concurrent chemoradiotherapy, with the intensity-modulated radiation field including the ipsilateral tongue base and tonsil. A CR was confirmed in December 2021 based on RECIST version 1.1 (**Figures 1B**, **2B**).

In February 2022, the patient developed dysphagia and hemoptysis. Head and neck MRI revealed a newly developed irregular mass measuring 40 mm × 47 mm in the right oropharyngeal region (**Figure 1C**). FOL showed a yellow-white lesion at the right tongue base extending beyond the midline, without invasion of surrounding oropharyngeal structures (**Figure 2C**). The disease was classified as progressive according to RECIST 1.1. Given the extent of tumor enlargement, poor performance status, and increased risk of airway obstruction, surgical resection was deemed high-risk and not pursued. After careful assessments of the patient’s performance status (KPS = 70), treatment tolerance, and tumor imaging, systemic chemotherapy with a weekly PCC regimen was initiated in February 2022. After two cycles of treatment, a significant reduction in tumor burden was observed on follow-up MRI and FOL. By June 2022, the patient achieved a CR (**Figures 1D**, **2D**) and resumed normal activities with excellent performance status (KPS = 100). There were no significant adverse events during the treatment process, except for leukopenia (2.33 × 10^9^/L) documented in July 2022.

IHC testing performed on a tumor biopsy sample revealed a positive PD-L1 result, CPS = 20. Given the PD-L1 positive status, the patient received cetuximab 400 mg fixed dose and tislelizumab 200 mg fixed dose intravenously on the first day of each 3-week cycle as maintenance therapy starting from September 2022. Serial evaluations, including MRI, CT, and FOL, revealed no evidence of tumor recurrence or progression. The patient remained in a good clinical condition (KPS = 100) with no new symptoms or significant adverse events. In January 2024, the patient developed mild joint pain and swelling in both upper and lower extremities, which were initially not evaluated. By April 2024, the symptoms had progressed to moderate joint pain with symmetrical swelling of the proximal interphalangeal, metacarpophalangeal, and wrist joints, accompanied by morning stiffness. A series of rheumatological investigations were performed in April 2024. Laboratory results were as follows: erythrocyte sedimentation rate (ESR), 15 mm/H; C-reactive protein (CRP), 4.57 mg/L. Autoantibody testing was positive for rheumatoid factor immunoglobulin M (RF IgM 32.4RU/ml), while RF IgA and RF IgG, anti-cyclic citrullinated peptide (CCP), anti-nuclear antibody (ANA), and human leukocyte antigen B27 (HLA-B27) were negative. Additionally, serum immunoglobulin M (IgM) levels were slightly elevated (3.12g/L vs. normal values of IgM range from 0.46 to 3.04g/L). The right knee joint MRI was performed using T1-weighted, T2-weighted fat-saturated, and contrast-enhanced sequences, revealing synovial thickening of the right knee joint, a small amount of joint effusion in the joint space, and no bone destruction. The diagnosis of ICI-IA was established per the 2023 Chinese Society of Clinical Oncology (CSCO) toxicity management guidelines for ICIs, following multidisciplinary consultation and exclusion of alternative causes of inflammatory arthritis (including paraneoplastic syndromes, prior chemo/radiation effects, cetuximab reactions, and infection). Corticosteroid therapy with oral prednisone acetate (1 mg/kg/day) was initiated, resulting in significant joint pain resolution after 4 weeks of therapy. The positive clinical response to steroid therapy further supported the diagnosis of ICI-IA.

Due to the severity of the irAE, therapy with ICI and cetuximab was discontinued in April 2024. Despite cessation of ICI therapy, mild inflammatory arthritis persisted but remained manageable and did not impair basic activities of daily living. Unfortunately, the instrumental activities of daily living are slightly impacted due to joint dysfunction. Follow-up MRI (**Figure 1E**) and FOL (**Figure 2E**) in April 2025 revealed no signs of tumor progression. The patient’s clinical course is summarized in **Table 1**.

**Table 1:** Timeline of the treatment course.

Timelines	Diagnostic evaluation	Treatments	KPS	Response evaluation
Aup.2021	unknown primary carcinoma of the head and neck	neck dissection and concurrent chemoradiotherapy	90	complete response
Feb.2022	a newly developed irregular mass measuring 40 mm × 47 mm in the right oropharyngeal region	multidisciplinary consultation	70	relpase
Feb.2022-Jun.2022	poor performance status, and increased risk of airway obstruction	weekly PCC regimen	100	complete response
Sep.2022	no clinical symptoms	cetuximab and tislelizumab	100	complete response
Apr.2024	G3 ICI-induced inflammatory arthritis	corticosteroid therapy and discontinuation of ICI and cetuximab	70	complete response
Jun.2024-Present	mildly impaired instrumental activities of daily living	follow-up	90	complete response

KPS, Karnofsky performance status; ICI, Immune checkpoint inhibitor.

## 3 Discussion

The National Comprehensive Cancer Network (NCCN) guidelines lack specific recommendations for maintenance therapy in patients with recurrent or metastatic head and neck squamous cell carcinoma (R/M HNSCC) who achieve good response following first-line treatment. In the present case, the patient experienced rapid disease progression after initial treatment, received weekly PCC regimen, and achieved a CR. Although cetuximab induced marked tumor shrinkage, maintenance cetuximab monotherapy failed to provide satisfactory survival benefits, as reported in the EXTREME trial (10). However, the TPExtreme trial (11) supported first-line TPEx regimen followed by second-line immunotherapy as a promising strategy for R/M HNSCC. In addition, results from two clinical trials (12, 13) demonstrated that combining PD-1 and EGFR blockade in R/M HNSCC yielded favorable ORR and OS compared with pembrolizumab monotherapy. Based on the above evidence, we initiated maintenance therapy with cetuximab and tislelizumab starting from September 2022. After 20 months of sustained CR, the patient experienced a grade 3 irAE, requiring discontinuation of ICI therapy.

IrAEs are a common occurrence in patients receiving ICI therapy, and they may affect virtually any organ system during or after treatment. Although the precise pathophysiological mechanisms remain unclear, emerging evidence suggests that irAEs may be mediated by immune system hyperactivation (14). This hyperactivated immune state, while implicated in the development of irAEs, may also enhance anti-tumor activity. Therefore, we hypothesize that irAEs may serve as an indirect indicator of T-cell activity and a potential biomarker of immunotherapy efficacy. Despite the potential clinical implications of this association, existing literature evaluating the predictive value of irAEs remains limited. In the present case, the patient with recurrent head and neck squamous cell carcinoma (HNSCC) achieved a CR to treatment with the anti-PD-1 agent tislelizumab. However, treatment was complicated by the development of ICI-IA, necessitating drug discontinuation. Notably, the patient remained in CR for over 12 months after cessation of ICI therapy, with no evidence of disease progression. This observation suggests that irAE occurrence may reflect sustained immune hyperactivation and could potentially serve as a potential biomarker for long-term immunotherapy efficacy, even after treatment withdrawal. Given these findings, large-scale, multi-center clinical studies are warranted to validate the prognostic value and clinical applicability of irAEs as predictive biomarkers of immunotherapy efficacy.

Among irAEs, cutaneous, gastrointestinal, and endocrine manifestations are most commonly reported. In contrast, rheumatic irAEs have a relatively lower incidence, but they remain clinically significant due to their potential impact on the quality of life. Clinically, rheumatic irAEs are categorized into musculoskeletal and non-musculoskeletal subtypes (15). Among these subtypes, ICI-induced musculoskeletal irAEs are more prevalent and primarily include inflammatory arthritis (IA), polymyalgia rheumatica/giant cell arteritis (PMR/GCA), and inflammatory myopathy (IM) (16, 17). Pooled data from three prospective studies (18–20) of ICI-treated patients revealed that the median onset time of musculoskeletal irAEs was approximately 3 months, with an overall incidence of 5–8%, while IA occurred in 2–3% of cases.

The pathogenesis of ICI-IA remains incompletely understood. Studies suggest that ICIs induce autoreactive B and T cell activation, leading to the production of autoantibodies and release of pro-inflammatory cytokines such as tumor necrosis factor-α (TNF-α), interleukin-6 (IL-6), and interleukin-17 (IL-17) (21). However, the role of autoantibodies in ICI-IA remains a subject of debate. Some studies have reported that anti-CCP was not detected in all ICI-IA patients and only a small proportion of ICI-IA patients test positive for RF and ANA (22, 23). Cappelli et al. reported that 11.4% of patients with ICI-IA tested positive for anti-RA33 autoantibodies, while these antibodies were consistently negative in ICI-treated patients without arthritis (24). In the present case, RF IgM was positive, while anti-CCP and other autoantibodies were negative, highlighting the heterogeneity of serological findings in ICI-IA. These findings suggest that certain autoantibodies may contribute to the ICI-IA pathogenesis; however, their diagnostic and prognostic value requires further investigation.

ICI-IA currently lacks a standardized clinical definition. It is typically characterized by joint stiffness—particularly in the morning—progressive pain, limited range of motion, and symptomatic relief with physical activity or heat application. Diagnostic evaluation should include assessment of inflammatory markers such as erythrocyte sedimentation rate (ESR) and C-reactive protein (CRP), autoantibody panels including antinuclear antibody (ANA), RF, and anti-CCP, as well as advanced imaging modalities such as US or MRI to detect synovitis, joint effusion, or erosive changes. Management of ICI-IA depends on the severity of symptoms. For grade 1 (G1) arthritis, non-steroidal anti-inflammatory drugs (NSAIDs) may provide adequate symptomatic control without the need to discontinue ICI therapy. In the present case, the patient initially developed mild joint discomfort approximately 16 months after initiation of ICI therapy, which was self-managed with NSAIDs. However, symptoms progressed within 3 months to severe polyarticular swelling and morning-exacerbated pain. After consultation with a rheumatologist and further testing, a diagnosis of grade 3 ICI-IA was confirmed. ICI therapy was discontinued, and corticosteroid therapy with oral prednisone acetate (1 mg/kg/day) was initiated. Although significant symptomatic improvement was observed after 4 weeks of treatment, joint dysfunction persisted, impairing the patient’s instrumental activities of daily living. For G3–G4 events unresponsive to corticosteroids (methylprednisolone) within 2 weeks of treatment, conventional synthetic disease-modifying antirheumatic drugs (DMARDs) should be considered, alongside temporary or permanent cessation of ICI therapy.

Notably, the patient in this case maintained a CR for over 1 year after cessation of ICI therapy, suggesting that the ICI-induced immune activation may elicit long-term antitumor effects even after cessation of treatment. This observation aligns with findings from two prospective studies in patients with various cancer types, where patients who developed musculoskeletal irAEs during ICI treatment exhibited significantly higher tumor response rates than those who did not (18, 20). In another study of 114 patients with metastatic HNSCC treated with anti-PD-1 antibodies (25), 60 irAEs were observed in 49 of 108 evaluable patients (45.4%), with the most frequently affected systems being dermatologic (21 of 60, 35.0%), musculoskeletal (15 of 60, 25.0%), and endocrine (14 of 60, 23.3%). Patients who experienced irAEs exhibited significantly improved ORRs (30.6% vs. 12.3%, p = 0.02), PFS (6.9 vs. 2.1 months, p = 0.0004), and OS (12.5 vs. 6.8 months, p = 0.0007) compared with those who did not. Multivariate analysis confirmed that irAE onset was independently associated with improved ORR (p = 0.03), PFS (p = 0.0009), and OS (p = 0.003). Low-grade irAEs (grades 1-2) are consistently associated with improved survival (OS: HR 0.57, 95% CI 0.43-0.75) based on the meta-analysis by Zhou et al. (8), while high-grade irAEs (grades 3-5) show increased ORRs but worse OS based on the meta-analysis by Hussaini et al. (9). In the present case, the development of grade 3 ICI-IA suggests a state of excessive immune activation. Even after discontinuation of ICI therapy, the patient remained in complete remission, suggesting that ICI-IA occurrence could serve as a reliable predictor of ICI efficacy in HNSCC. However, further studies are warranted to determine whether specific irAE subtypes, such as musculoskeletal irAEs, possess independent prognostic value in HNSCC.

Given the frequent occurrence of musculoskeletal symptoms among cancer patients (26, 27), a comprehensive differential diagnosis is essential to distinguish between joint pain from immune-related inflammatory arthritis and other etiologies. Despite the relatively low incidence of immune-related arthritis in cancer patients undergoing immunotherapy, the potential for severe joint dysfunction and impairment of patients’ quality of life underscores the need for early involvement of experienced rheumatologists and multidisciplinary collaboration to facilitate accurate diagnosis, prompt intervention, and comprehensive risk-benefit assessments for ICI continuation, tailored to individual patients. This is particularly important because rheumatologic irAEs often follow a chronic or relapsing course distinct from both non-rheumatic irAEs and classical autoimmune diseases and may persist even after discontinuation of immunotherapy (17, 28). Therefore, personalized management strategies and long-term follow-up are essential for optimizing clinical outcomes.

## 4 Conclusion

This report describes a patient with advanced UPCHN who achieved long-term CR from treatment with tislelizumab. Despite the occurrence of severe ICI-IA, necessitating discontinuation of ICI therapy, the patient maintained CR for over 1 year after cessation of treatment. These findings suggest that irAEs may serve as a potential biomarker of ICI efficacy. However, further validation in large-scale, prospective clinical studies is warranted to establish the prognostic significance and clinical applicability of irAEs.

## Data Availability

The original contributions presented in the study are included in the article/supplementary material. Further inquiries can be directed to the corresponding authors.
